# High-Sensitivity CO_2_ Sensor Based on a Graphene Oxide Coated Long-Period Fiber Grating

**DOI:** 10.1021/acsomega.5c00184

**Published:** 2025-05-28

**Authors:** Anne C. P. Fernandes, Nayton C. Vicentini, Matheus S. Couto, Giovanni R. Carvalho, Benjamin Fragneaud, Cristiano Legnani, Indhira O. Maciel, Renato Luiz Faraco Filho, João Victor de Castro Nascimento, João Pedro Emanuel Ferreira, Felipe Barino, Diogo Coelho, Alexandre Bessa dos Santos, Welber G. Quirino

**Affiliations:** † Nanosciences and Nanotechnology Group—Nano, Physics Department, Federal University of Juiz de Fora, 36036-900 Juiz de Fora, Brazil; ‡ Instrumentation and Telemetry Laboratory—LiTel, Electrical Circuit Department, Federal University of Juiz de Fora, 36036-900 Juiz de Fora, Brazil

## Abstract

This study presents the development and characterization of a novel carbon dioxide (CO_2_) sensor based on graphene oxide (GO)-coated long-period fiber grating (LPFG). The structural and chemical properties of GO were analyzed using Raman spectroscopy and scanning electron microscopy (SEM), revealing a defective structure with a high degree of oxidation and significant surface roughness, which enhances gas adsorption capabilities, making it highly suitable for CO_2_ detection. The sensor’s performance was evaluated across CO_2_ concentrations ranging from 5 to 65%. The operational principle of the sensor is based on changes in the resonance wavelength induced by variations in the refractive index of the GO coating as it interacts with CO_2_ molecules. Results indicate a notable sensitivity of 0.0643 nm/% and low hysteresis during adsorption and desorption processes, affirming its stability and reliability. Additionally, the sensor demonstrated a strong linear fit of approximately 96% in adsorption and desorption cycles (5–65 and 65–5%). These findings underscore the significant potential of the GO-coated LPFG sensor for practical CO_2_ sensing applications, offering advantages such as immunity to electromagnetic interference and ease of integration into remote sensing technologies.

## Graphical abstract

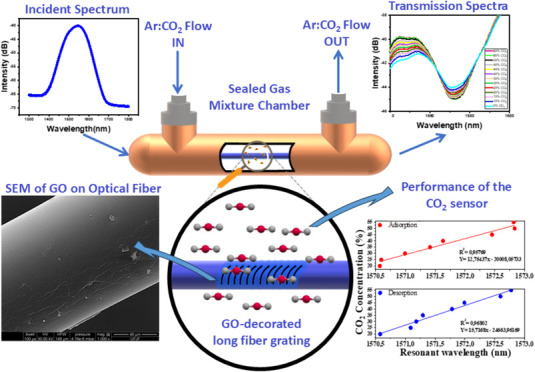

## Introduction

The detection of carbon dioxide (CO_2_) is of paramount importance due to the numerous environmental and health problems this gas can cause. Additionally, CO_2_ detection plays a fundamental role in various industrial sectors, such as the food industry, where it is essential for ensuring product quality control;^1,2^ the oil industry, where it is utilized to monitor oil wells;^3^ the chemical industry, where it aids in controlling chemical processes; and the medical field, where it assists in disease diagnosis^4^ and significantly contributes to accident prevention.^5^ Consequently, precise and noninvasive CO_2_ monitoring is required, especially under unfavorable conditions of humidity, pressure, and temperature, such as those found in oil wells or deep mines.^6,7^ The primary commercially available sensors for CO_2_ detection are nondispersive infrared (NDIR) or semiconductor devices.^8^ However, these conventional sensors face limitations, including high cost, significant weight and size, low durability, and susceptibility to electromagnetic interference. Thus, the development of fiber optic sensors has gained attention in recent years, as they offer compact size, immunity to electromagnetic interference, integration of multiple sensors into a single system, remote monitoring capabilities, low cost, and applicability in hostile environments.^9,10^ Long-period fiber grating (LPFG) sensors have been extensively studied for gas sensing applications due to their high sensitivity to changes in the surrounding refractive index.^11^ An LPFG sensor essentially consists of periodic modulations on the fiber core, which modify its refractive index.^12^ These periodic disturbances enable the fundamental mode to interact with cladding modes, altering the refractive index and, consequently, the transmission spectrum, thereby enhancing system sensitivity.^13^ However, LPFGs fabricated in uncoated optical fibers exhibit inherent resilience to gas variations.^1^ Since silica optical fibers are chemically inert, fiber-optic chemical sensing typically relies on converting analyte information into variations in measurable parameters, such as the change in the resonance wavelength.^1,14–17^

To improve the sensitivity of LPFG sensors, the fiber was coated with a nanomaterial called graphene oxide (GO). This nanomaterial was chosen because, as reported in the literature, it exhibits superior sensitivity for detecting gases under ambient conditions.^18,19^ Graphene oxide is a nanomaterial derived from graphene, characterized by a two-dimensional (2D) structure with sp^2^ and sp^3^ hybridization and enriched with various oxygen-containing functional groups, such as hydroxyl (OH), epoxy (C–O–C), carboxylic acid (HO-C=O), and carbonyl (C=O), within a single layer of graphene.^20,21^ Graphene oxide has numerous applications, largely due to the presence of oxygenated groups that contribute to its properties and functionalities.^22^ In the context of sensors, these functional groups are key in determining the specific sensitivity of GO to target gases. However, this wide range of possibilities can reduce selectivity, as cross-detection of multiple agents may occur. Nonetheless, studies by Akhter et al.,^18^ Shaban et al.,^19^ and Zhao et al.^23^ have demonstrated that GO exhibits a notable selectivity toward CO_2_ compared to other gases. Additionally, the high hydrophilicity of GO makes humidity a significant factor in gas detection, influencing both adsorption and sensor response, as highlighted in studies,^24–28^ which indicate that water molecules compete with target gases for adsorption sites, leading to signal drift and reduced selectivity. Additionally, the LPFG is highly sensitive to temperature variations,^29,30^ further complicating the sensor’s response. Consequently, one could infer that environmental measurements with this sensor may be compromised by its sensitivity to temperature and humidity. However, the authors in^1^ have addressed and resolved this cross-sensitivity issue in optical sensing by employing artificial neural network methods. This allows for the filtering of CO_2_ signals from other undesirable parameters in practical applications. It is important to note, however, that while this study was primarily focused on CO_2_ detection and we acknowledge that graphene oxide (GO) exhibits sensitivity to a wide range of gases and volatile organic compounds (VOCs), our sensor was not tested under environmental conditions. Instead, our primary goal was to demonstrate its excellent sensitivity, reproducibility, and overall performance. For this purpose, the samples were tested in a sealed gas chamber, as detailed in the Experimental Methods section. For future applications, we anticipate that signal filtration methods or other approaches could be employed to address potential cross-sensitivity issues in real-world environments. Specifically for CO_2_ sensors, the functional groups in GO facilitate interactions with CO_2_ molecules through both covalent and noncovalent bonds.^31,32^ The interaction between carbon dioxide and graphene oxide occurs as CO_2_ molecules bind to the electron-rich oxygen groups of graphene oxide. Additionally, the oxygen molecules in CO_2_ can interact with hydrogen-containing groups located at the edges of GO.^33^ Another important property of GO is that its oxygenated groups enable dispersion in a wide range of solvents and at varying concentrations, facilitating its application as a coating on LPFG gratings. The high surface area of GO^34^ also enhances its molecular adsorption efficiency.^18^ Furthermore, to ensure improved adhesion of GO to the LPFG surface, (3-Aminopropyl) triethoxysilane (APTES) is employed. According to Pokhrel et al., APTES increases the adsorption capacity of GO for CO_2_ by approximately 36%.^35^ This enhancement is attributed to the presence of hydroxyl groups, which exhibit high surface energy, allowing rapid interaction and covalent bonding with siloxane groups (−Si-O−), thereby ensuring more stable functionalization and improving CO_2_ capture selectivity. Additionally, APTS functionalizes the graphene oxide surface by introducing amine groups (−NH_2_), which strongly interact with CO_2_ molecules through Lewis acid–base interactions, further enhancing adsorption capacity. These amine groups can also undergo nucleophilic attack on the carbon atom of CO_2_. Numerous studies in the literature have demonstrated that the interaction between –NH_2_ groups and the adsorbent, facilitated by existing Lewis and Brønsted acidic sites, further improves CO_2_ adsorption and selectivity.^36–39^ Moreover, the functionalization of GO with APTES also renders it hydrophobic,^37,40^ helping to mitigate the cross-sensitivity of the sensor to humidity, as nonfunctionalized GO remains highly sensitive to moisture.

## Experimental Methods

### Sensor Fabrication

The sensor fabrication process comprises three main steps: the creation of the LPFG, the synthesis of GO, and the deposition of GO onto the LPFG.

### Fiber Preparation

The LPFG sensor was fabricated using the electric arc technique on a single-mode fiber (SMF-28).^41^ We employed the same manufacturing setup and equipment as described by Filho et al.^30^ This process involves creating periodic deformations in the fiber cladding through an electric arc, with a consistent spacing of 500 μm. The ends of the LPFG were mounted on a Teflon support to ensure the fiber remained straight and stationary throughout the process. This setup prevents fiber breakage, ensures uniform GO deposition, and stabilizes the LPFG/GO system during measurements. To remove organic contaminants, the grating region of the fiber underwent a cleaning procedure. This involved immersing the recorded region sequentially in an acetone solution, a hydrochloric acid (HCl,1M) solution at room temperature, and finally rinsing it with deionized water (DI).

### Silanization

To improve the adhesion of GO to the LPFG surface, a silanization procedure was performed. The process began with an alkaline treatment, where the sensor was immersed in a 1 M NaOH solution for 1 h at room temperature to increase the number of hydroxyl groups (−OH) on the fiber surface. After thorough rinsing with DI water, the LPFG was immersed in a 5% (v/v in ethanol) (3-aminopropyl) triethoxysilane (APTES) solution for 2 h at room temperature, forming a Si–O–Si bond on the surface. To complete the silanization process, the sensor was washed with ethanol to remove unbound monomers.

### GO Preparation

The preparation of GO generally involves modifications of the traditional Hummers method.^24,42,43^ In this study, GO was synthesized using the Hummers method with two oxidation steps and extended oxidation times to produce nanosheets with a high degree of oxidation and exfoliation, following the methodology previously developed by Lima et al.^44^

To synthesize GO, natural graphite flakes were oxidized over 120 h using strong oxidizing agents such as sulfuric acid, potassium permanganate, and sodium nitrate. A second oxidation process was then conducted for 3 h with permanganate ions alone. Following the oxidation steps, the resulting material was purified through sequential rinses with acid solutions to remove metal traces and reaction byproducts. These rinses continued with deionized water until the GO dispersions reached a pH of 6–7. Finally, the material was dried and dispersed in an aqueous medium using advanced ultrasound techniques, yielding a solution with a concentration of 1 mg/mL for incorporation onto the LPFG. Figure 1 displays images of the sequential steps in the synthesis of the GO solution.

**Figure 1:**
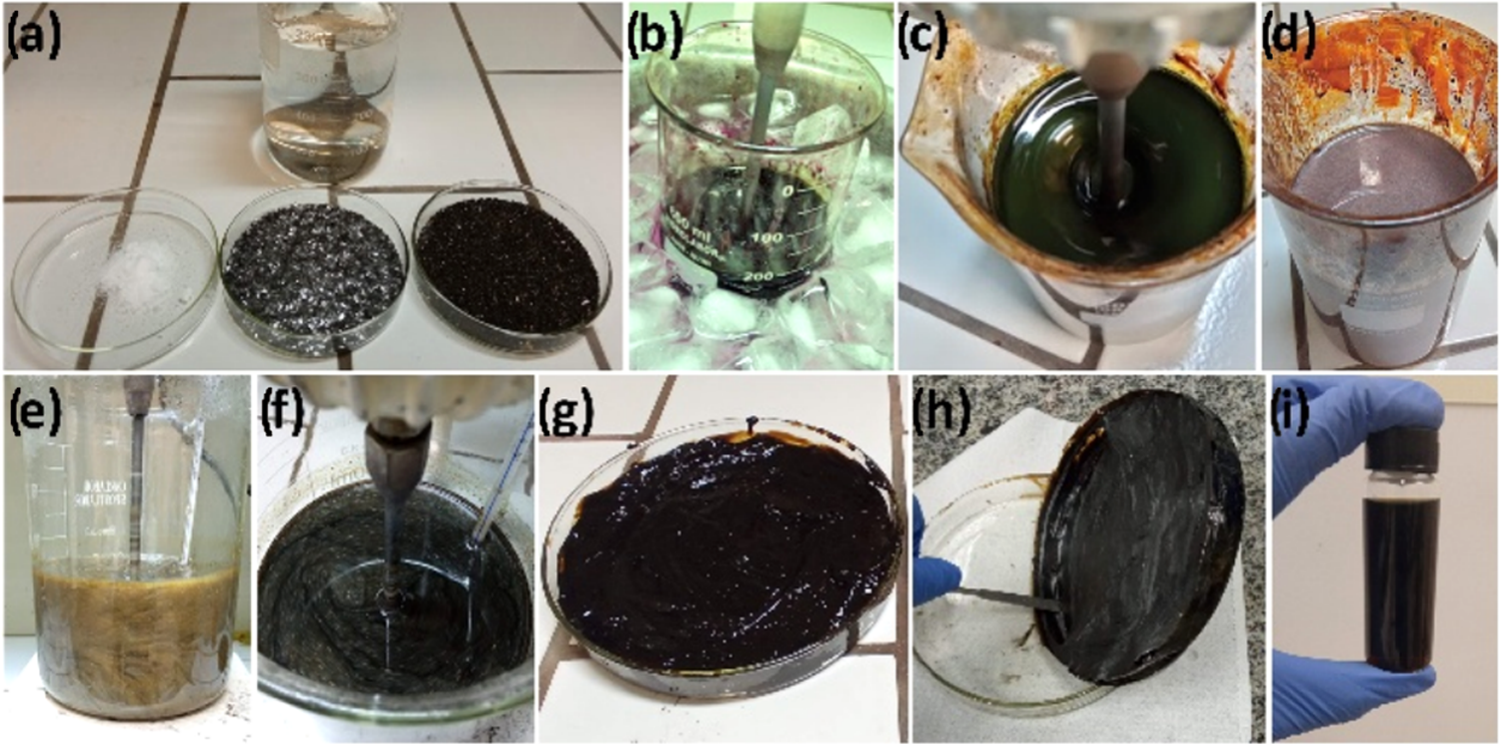
Sequential steps in the synthesis of the GO solution. (a) Reagents used: sulfuric acid, graphite flakes (Sigma-Aldrich), potassium permanganate, and sodium nitrate. (b) Ice bath during reagent mixing. (c) Solution after 24 h of reaction. (d) Solution after 120 h of stirring at room temperature, marking the end of the first oxidation stage. (e) Diluted solution with 5 wt % H_2_SO_4_ at approximately 80 °C (second oxidation stage). (f) Solution after 3 h of oxidation. (g) Solid paste obtained after the purification step. (h) Material after the drying stage. **(i)** GO solution after dispersion in DI water using an ultrasonic processor.

### Sensor Functionalization

The sensor was functionalized using the dip-coating technique, immersing it three times in a GO solution (1 mg/mL in DI water) at 50 °C. This procedure follows previous studies in the literature, which recommend multiple immersions to ensure the formation of a uniform film on the sensor surface.^17,45–47^ After coating, the sensor was left to stabilize at room temperature before measurements in the test chamber. Sensors with fewer coatings were also tested but did not provide a satisfactory signal.

### Operating Principle

For an LPFG, the resonance wavelength, λ_res_^m^ which describes the coupling between the fundamental core mode and the *m*th evanescent cladding mode, can be expressed as eq 1.^48^

1 $$\lambda_{res}^{m}=\left(n_{eff\_co}-n_{eff\_cl}^{m}\right)\Lambda \left(m=1,2,\cdot \cdot \cdot \right)$$

where λ is the grating period and the *n*_eff_co_ and *n*_eff_cl_^m^ represent the effective refractive index of the core and cladding modes, respectively. The cladding mode is influenced by the thin film coating of GO on the grating surface, forming a four-layer waveguide consisting of the core, cladding, coating, and surrounding media. As GO absorbs CO_2_ molecules, the refractive index of the coating layer changes, altering the evanescent wave. This results in a variation in *n*_eff_cl_^m^ leading to a shift in the LPFG transmission spectrum. The LPFG’s attenuation rate, i.e., transmissivity at resonance, is described by the following eq 2 ^49^

2 $$T_{m}=1-\mathrm{si}n^{2}\left(k_{m}L\right)$$

where *k*_m_ is the coupling coefficient for the *m*th cladding mode, and *L* is the length of the LPFG. The coupling coefficient depends on the overlap integral between the core and cladding modes, the refractive index of the core and cladding (*n*_co_ and *n*_cl_), *n* ^m^, and the grating period. Since the coating layer’s refractive index changes due to the adsorption of CO_2_ molecules by the GO, this modifies the evanescent wave and *n* ^m^. Consequently, the coupling coefficient also changes, resulting in visible variations in the intensity of the sensor’s loss bands.^50^

The sensing mechanism of the graphene oxide (GO)-coated long-period fiber grating (LPFG) sensor is based on the interaction between the refractive index of the surrounding medium and the attenuation characteristics of the optical signal transmitted through the fiber. Since the attenuation at λ_res_^m^ is caused by scattering at the cladding and surrounding media interface, the refractive index of the surrounding medium affects the scattering properties. Moreover, the effective refractive index of the cladding *n*_eff_cl_^m^ is influenced by the surrounding refractive index (SRI), leading to a shift in λ_res_^m^ ^51^

3 $$\frac{d\lambda_{res}^{m}}{dn_{sur}}=-\lambda_{res}\frac{\frac{d\lambda_{res}}{d\Lambda }}{n_{eff\_co}-n_{eff\_cl}^{m}}\frac{u_{m}^{2}\lambda_{res}^{3}n_{sur}}{8\pi r_{cl}^{3}\left(n_{eff\_co}-n_{eff\_cl}^{m}\right){\left(n_{cl}^{2}-n_{sur}^{2}\right)}^{3/2}}$$

where *u*_m_ is the zeroth-order Bessel function *m*-th root, *r*_cl_ and *n*_cl_ is the cladding radius and refractive index, respectively.

In this work, the fundamental sensing mechanism relies on the interplay between the refractive index of the surrounding environment and the attenuation properties of light transmitted through the optical fiber. Specifically, the GO coating on the LPFG interacts with CO_2_ molecules, inducing a change in the coating’s refractive index. This interaction occurs as CO_2_ molecules bind to the electron-rich oxygen functional groups of graphene oxide, while the oxygen atoms in CO_2_ can interact with hydrogen-containing groups located at the edges of GO. This variation in refractive index alters the evanescent wave and the coupling coefficient within the LPFG, resulting in a detectable shift in the transmission spectrum. Consequently, by monitoring these spectral changes, the sensor effectively quantifies variations in CO_2_ concentration.

## Experimental Setup

To validate this operating principle, calibrate, and characterize the sensor’s performance, the sensor was placed inside a gas chamber designed by the authors. This chamber was connected to a gas system containing CO_2_ and Argon (Ar). The optical fiber was terminated with FC/APC connectors outside the chamber and connected to an optical spectrum analyzer (OSA–Anritsu MS9740B) to monitor the LPFG transmission spectrum under different CO_2_ concentration. The experimental setup is shown in Figure 2.

**Figure 2:**
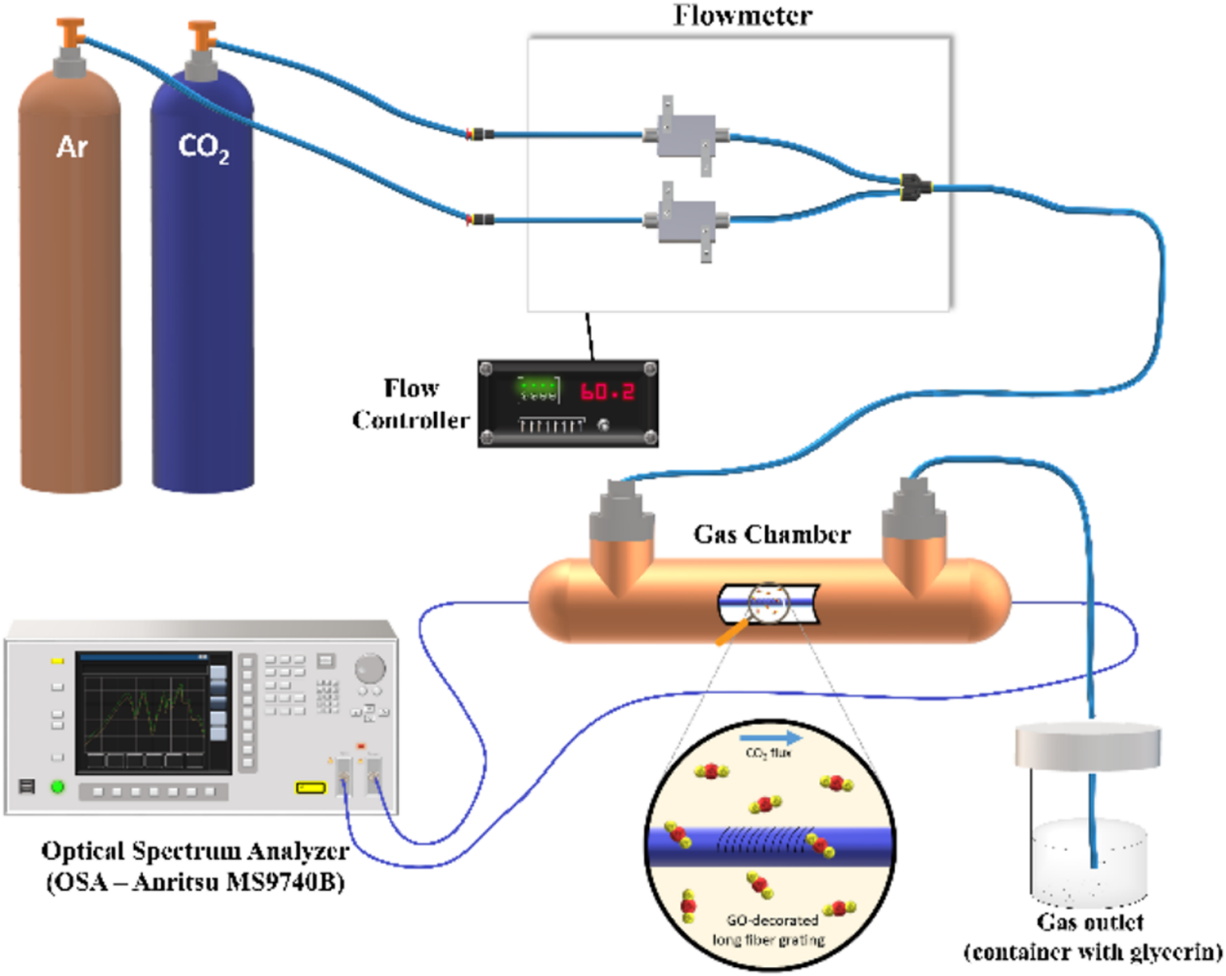
Experimental setup for carbon dioxide sensing.

The experiment utilized varying CO_2_ concentrations in Argon (Ar) to simulate detection under real-world conditions. The CO_2_ concentration ranged from 5 to 65%, with increments of 5%. Prior to starting the experiment, the chamber was purged with Ar to ensure the removal of any moisture. Additionally, the temperature was maintained constant at 25 °C throughout the entire experimental procedure. To adjust the gas concentration, a gas mixer with different flow controls was used to generate various gas samples in the sealed gas chamber. To obtain the appropriate concentration, the flow ratio of CO_2_ to Ar was adjusted using control software. The gas flow rates during the experiment were measured in Standard Cubic Centimeters per Minute (sccm), maintaining a constant total flow rate of 300 sccm. For instance, a 5% CO_2_ concentration was achieved by mixing 15 sccm of CO_2_ with 285 sccm of Ar.

## Results

### GO Chemical and Structural Characterizations

In Figure 3, the Raman spectra measurements are presented for GO and for a GO-coated optical fiber. The measurements were performed using a Bruker Senterra spectrometer with a 532 nm laser excitation at a power intensity of 0.2 mW. Raman spectroscopy is commonly employed in carbon-based material studies^52^ as it provides significant information about graphene oxide (GO) sheets and their nanostructures. In the spectra, two intense and well-defined peaks, D and G, are observed at approximately 1340 and 1600 cm^–1^, respectively. Additionally, three less intense peaks, 2D, D + G, and D + D′, are visible at 2720, 2920, and 3150 cm^–1^, respectively. The D peak is attributed to local defects and disorder in GO, caused by the attachment of carbonyl (C=O), hydroxyl (C–OH), epoxy (C–O–C), and carboxylic acid (HO–C=O) groups on the carbon basal plane and edge.^43,53^ The intensity of this peak decreases with increasing graphene crystallinity and a reduction in the number of defects.^54^ The G peak corresponds to the first order scattering of the E_2g_ mode of sp^2^-hybridized carbon atoms and reflects the quality of the graphitic structure.^55^ Another key metric derived from the spectra is the intensity ratio of the D and G bands (*I*_D_/*I*_G_),^56^ which provides insight into the material’s level of disorder. In this study, the *I*_D_/*I*_G_ ratio was calculated as 1.18, indicating a high degree of disorder. From this ratio, the distance between defects was estimated to be approximately 1.3 nm, further confirming the high level of disorganization in the material. Moreover, the intensity of the 2D peak is used to estimate the number of layers and the degree of exfoliation in GO. In this case, the results suggest fewer than three layers, highlighting significant exfoliation. This high level of disorder and exfoliation on the fiber surface is crucial for enhancing gas sensing performance.

**Figure 3:**
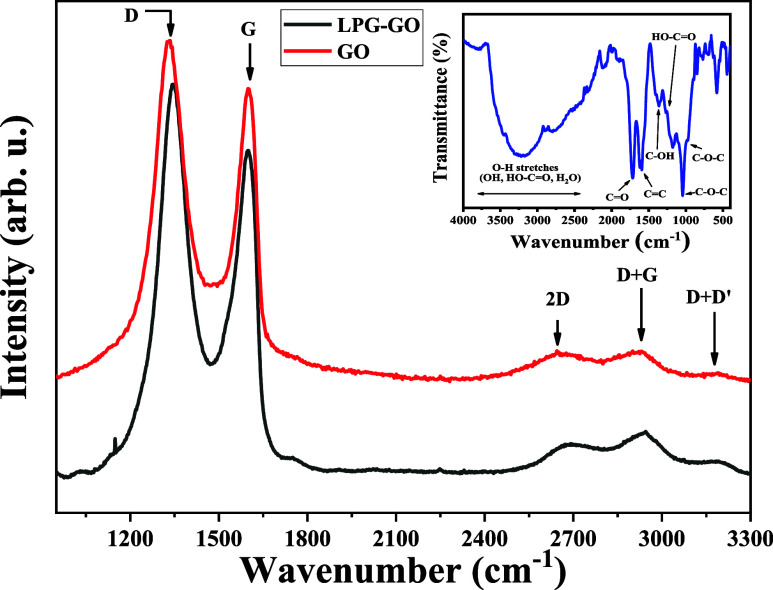
Raman spectrum of graphene oxide obtained with a laser excitation wavelength of 532 nm. The inset presents the FT-IR spectrum of the same sample, highlighting the main functional groups.

Additionally, the Raman spectrum of a GO-coated optical fiber is also presented in Figure 3, showing that the characteristic bands (D, G, 2D, D + G, and D + D′) remain consistent with those observed for the GO film. This observation confirms that the GO is well adhered to the fiber surface, preserving its structural and chemical characteristics.

The inset of Figure 3 presents the Fourier transform infrared (FT-IR) spectra of GO, highlighting a broad band at 3700–3000 cm^–1^ attributed to O–H stretching, with contributions from carboxylic groups and overlapping C–H stretching modes (3100–2600 cm^–1^). The band at 1720 cm^–1^ corresponds to C=O stretching, while the band at 1620 cm^–1^ is associated with C=C stretching within the GO structure. Bands at 1400 and 1280 cm^–1^ are related to C–OH bending and stretching, and those at 1040 and 980 cm^–1^ correspond to C–O–C stretching and out-of-plane bending modes.^57,58^

The surface morphology of the GO-deposited fiber was characterized using a Scanning Electron Microscope (SEM), specifically the FEI Quanta 250 model, operated at an acceleration voltage of 30 kV and with a 40 nm Au predeposition. In Figure 4 (1000× magnification), (a) LFPG surface without coating and (b) the SEM image the SEM image reveals that the GO deposition method produces a uniform coating, indicating successful deposition across the LPFG surface. In Figure 5 (5000× magnification), (a) LFPG surface without coating and (b) an enlarged SEM image of the same area reveals a surface with prominent texture. This textured surface contributes to an increased effective area, which is a beneficial feature for this type of sensor. Although the SEM does not directly measure roughness, the observed surface characteristics indicate that the deposition method effectively meets the morphological^59^ requirements for the sensor.

**Figure 4:**
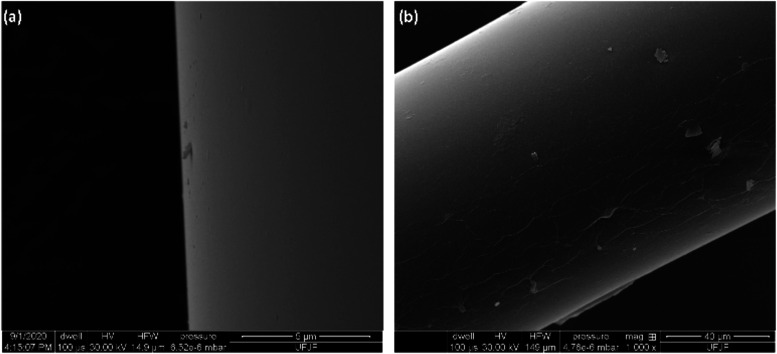
SEM image at 1000× magnification (a) of the core surface of an LPFG fiber surface without coating and (b) surface of an LPFG fiber coated with GO.

**Figure 5:**
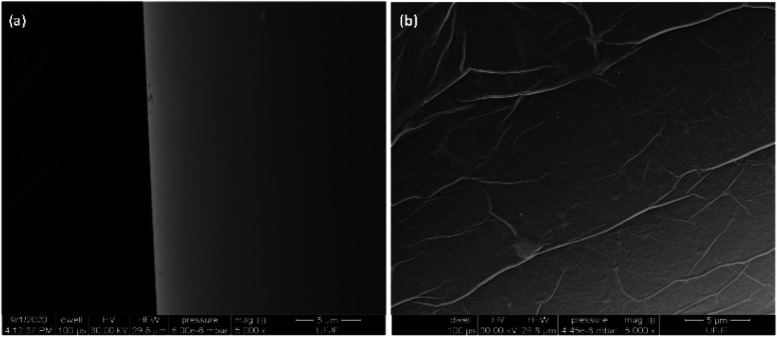
SEM image at 5000× magnification (a) of the core surface of an LPFG fiber surface without coating and (b) surface of an LPFG fiber coated with GO, the ripples of the GO nanosheets.

### Sensor Results

Figure 6 illustrates the characteristics of the LPFG sensor coated with GO for CO_2_ detection within the concentration range of 5 to 65%. Analyzing the transmission spectra of the resonant band shown in Figure 6 reveals that during the adsorption process, an increase in CO_2_ concentration induces a slight shift of the resonant band (centered at 1570 nm) toward longer wavelengths. Conversely, as depicted in Figure 7, during the desorption process, the transmission spectra exhibit behavior opposite to that observed during adsorption: as the CO_2_ concentration decreases, the resonant wavelengths undergo a red shift. As previously discussed, the shifts in the resonance bands of the sensor are attributed to variations in the refractive index, which result from the interactions between gas molecules and the active sites of the GO.^60^ Adsorbed gas molecules act as electron donors; as their concentration increases, electrons are transferred to the valence band of the GO. This electron transfer reduces the density of holes and increases the electron concentration.^6,18,61^ Consequently, the boundary conditions for light propagation are modified, leading to changes in the transmission spectrum.

**Figure 6:**
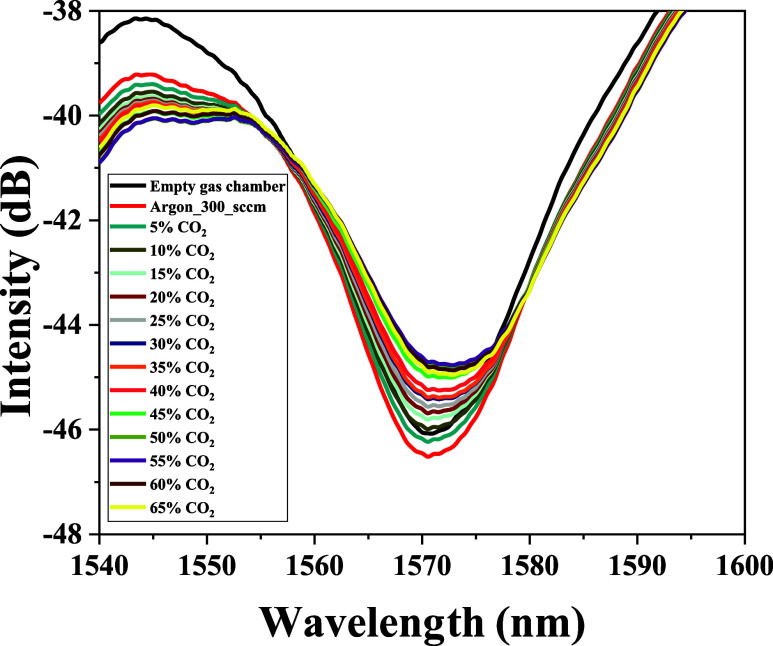
Transmission spectra of an LPFG coated with GO during CO_2_ adsorption at concentrations ranging from 5 to 65%.

**Figure 7:**
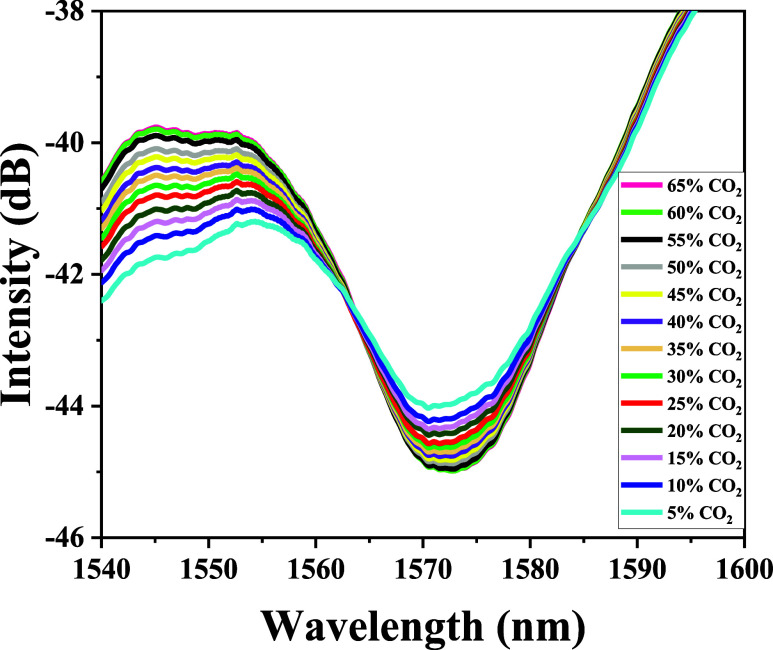
Transmission spectra of an LPFG coated with GO during CO_2_ desorption at concentrations decreasing from 65 to 5%.

The experimental results further confirm the inherent insensitivity of uncoated LPFGs to gas concentration variations, as shown in Figure 8 (blue dots). This behavior stems from the chemical inertness of silica optical fibers, which limits direct interactions with CO_2_ molecules. Consequently, uncoated LPFGs exhibit minimal shifts in resonance wavelength. In contrast, when coated with GO, the sensor demonstrates a clear and measurable response to increasing CO_2_ concentrations (red dots in Figure 8), validating the role of the functionalized layer in enhancing gas adsorption and interaction. This effect can be attributed to the presence of oxygen-containing functional groups in GO, which facilitate CO_2_ adsorption through physisorption and chemisorption mechanisms. As a result, the resonance wavelength shift observed in GO-coated LPFGs is significantly more pronounced, confirming the improved sensitivity and effectiveness of the functionalized sensor in detecting gas variations.

**Figure 8:**
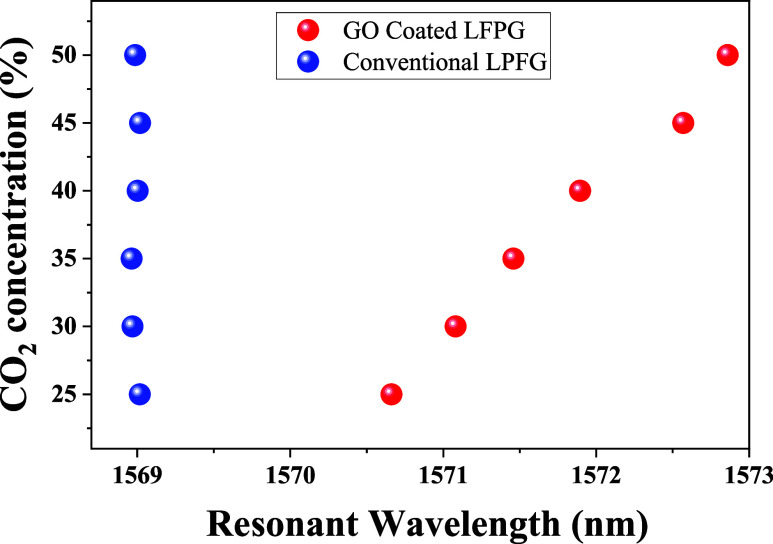
Resonant wavelength variation with increasing CO_2_ concentrations for uncoated (conventional) and coated LPFGs.

To evaluate the performance of the CO_2_ sensor, its sensitivity (*S*) was calculated using eq 4 ^62^

4 $$S\left(\%\right)=\left(\frac{\lambda_{\max}-\lambda_{\min}}{\Delta \left(\%\right)}\right)$$

Where Δ­(nm) and Δ (%) represent the variation in the resonant wavelength and the change in CO_2_ concentration between the maximum (65%) and minimum (5%) levels, respectively. The eq ([Disp-formula eq4]) is widely accepted in the literature for sensitivity calculations in similar optical sensing systems.^63–65^ However, as observed in the adsorption and desorption process illustrated in Figure 9, the sensor exhibited a detection dead zone within CO_2_ concentration ranges of 5 to 20% and 55 to 65%. This behavior can be attributed to two main factors: at low CO_2_ concentrations (<20%), the limited availability of CO_2_ molecules results in insufficient interaction with the oxygen-containing functional groups of GO, leading to a weak or delayed sensor response. Conversely, at high concentrations (>55%), the sensor reaches saturation, meaning that all active sites on the GO surface and between its layers are fully occupied, preventing further adsorption and making the sensor unresponsive to additional CO_2_ increases.^66–69^ Consequently, the analysis focused on the CO_2_ concentration range between 20 and 55% during both adsorption and desorption process. Based on this analysis, the sensor demonstrated sensitivity of approximately 0.0643 nm/%, for adsorption, and 0.0629 nm/%, for desorption. Furthermore, as shown in Figure 9, the sensor exhibited low hysteresis during both the adsorption and desorption processes, confirming the device’s stability within this concentration range.

**Figure 9:**
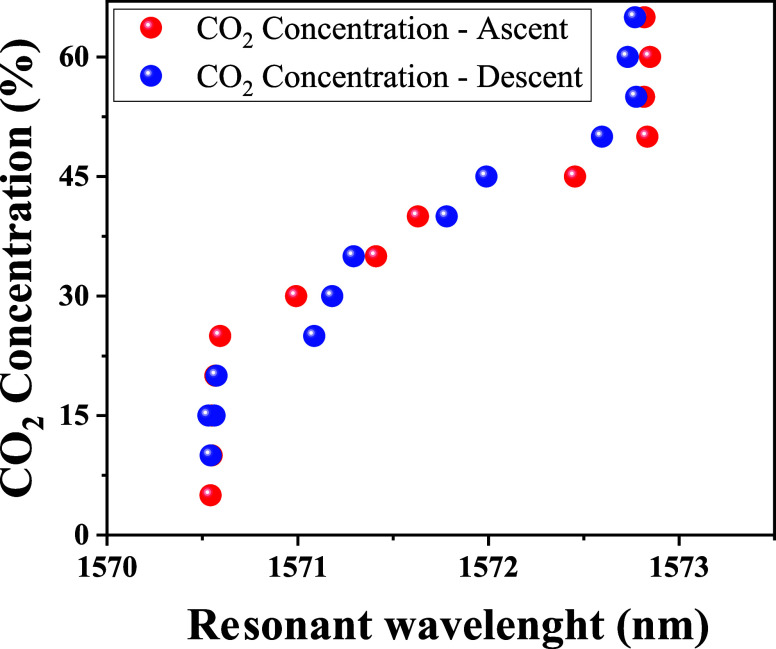
Shift in resonant wavelength with increasing and decreasing CO_2_ concentrations in the range of 5 to 65% for the LPFG sensor coated with GO.

The sensor demonstrated excellent linearity during both adsorption and desorption processes, with linear regression coefficients of approximately 96 and 97%, respectively, within the concentration ranges of 20–55 and 55–20%, as illustrated in Figure 10.

**Figure 10:**
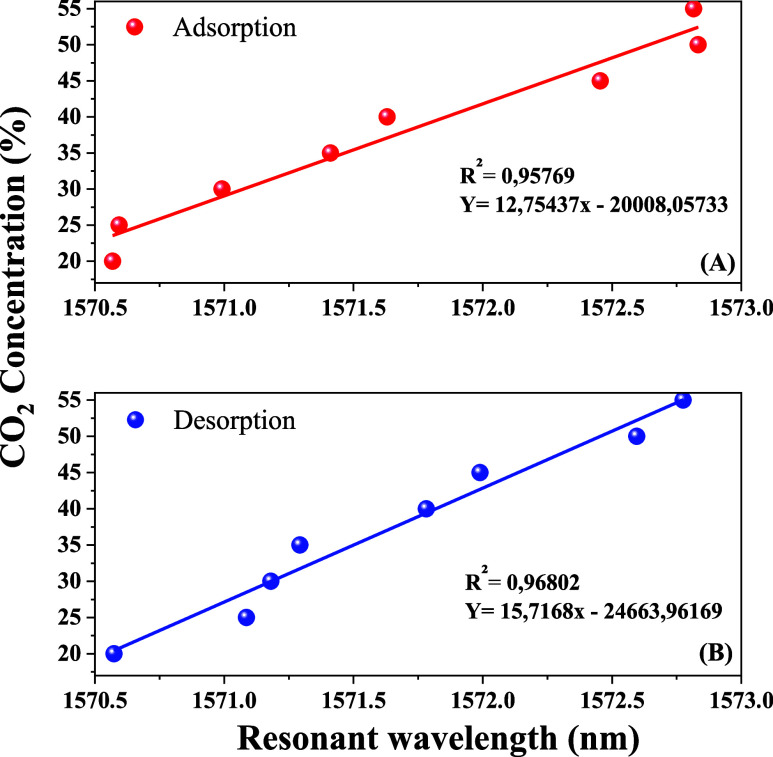
Wavelength changes with variations in CO_2_ concentration: (A) increasing from 20 to 55% and (B) decreasing from 55 to 20%.

## Conclusions

This study aimed to report on the development and performance of a GO-coated LPFG sensor for CO_2_ detection. Raman spectroscopy and SEM analyses confirmed that GO exhibits a defective structure, a high degree of oxidation, and a large surface area with a rough morphology, all of which make it highly suitable for CO_2_ detection. These structural characteristics contributed to the sensor’s excellent performance during both adsorption and desorption processes, demonstrating sensitivities of 0.0643 and 0.0629 nm/%, respectively. Additionally, the sensor exhibited high linearity, with regression coefficients of approximately 96% for adsorption and 97% for desorption. This study demonstrated that the GO-coated LPFG sensor holds significant potential for CO_2_ sensing applications. Its advantageous properties, including immunity to electromagnetic interference, multiplexing capability, and ease of integration with remote sensing systems, further enhance its appeal across various application fields.
